# Definition of an adapted cut-off for determining low lean tissue mass in older women with obesity: a comparison to current cut-offs

**DOI:** 10.1038/s41598-022-21258-5

**Published:** 2022-10-07

**Authors:** Laurent Maïmoun, Chris Serrand, Thibault Mura, Eric Renard, David Nocca, Patrick Lefebvre, Vincent Boudousq, Antoine Avignon, Denis Mariano-Goulart, Ariane Sultan

**Affiliations:** 1 Département de Médecine Nucléaire, CHRU Montpellier, Montpellier, France; 2 U1046 INSERM, UMR9214 CNRS, Physiologie et Médecine Expérimentale du Cœur et Des Muscles (PHYMEDEX), University of Montpellier, CHRU Montpellier, Montpellier, France; 3 Département d’Information Médicale, CHRU Nîmes, Nîmes, France; 4 Departement d’Endocrinologie, Diabète, Nutrition, Hôpital Lapeyronie, CHRU Montpellier, Montpellier, France; 5 Département de Chirurgie Digestive, CHRU Montpellier, Montpellier, France; 6 Département de Médecine Nucléaire, CHRU Nîmes, Nîmes, France; 7 Département Endocrinologie, Nutrition, Diabète, Equipe Nutrition, Diabète, CHRU Montpellier, Montpellier, France; 8 Desbrest Institute of Epidemiology and Public Health, IDESP, UMR UA11 INSERM, University of Montpellier, Montpellier, France; 9 Département de Biophysique, Service de Médecine Nucléaire, Hôpital Lapeyronie, Université de Montpellier, 371, avenue du Doyen Gaston Giraud, 34295 Montpellier, France

**Keywords:** Diseases, Endocrinology

## Abstract

The prevalence of sarcopenia in patients with obesity varies according to the definition used. The purpose of our study was to: (i) determine the prevalence of sarcopenia in terms of lean tissue mass in older women with obesity using the current cut-offs, (ii) redefine a specific cut-off for low lean tissue mass (LLTM), and (iii) re-determine the prevalence of LLTM using this new cut-off. Appendicular lean mass (ALM) and the ALM index [ALM/height^2^: ALMI(h^2^)] and ALMI/body mass index [ALMI(BMI)] were determined in 791 women with or without obesity. LLMM prevalence was calculated using the current cut-offs: EWGSOP2: ALM < 15 kg and ALMI(h^2^) < 5.5 kg/m^2^; FNIH: ALM < 15.02 kg and ALMI(BMI) < 0.51; and IWGS: ALMI(h^2^) < 5.67 kg/m^2^ and cut-offs newly determined from data provided from young women with obesity. ALM, ALMI(h^2^) and ALMI(BMI) were lower in older compared to young obese women. Using the current cut-offs, a wide distribution of LLTM prevalence (0 to 29.2%) was observed. When the newly determined cut-offs were applied – i.e., ALM < 18.51 kg; ALMI(h^2^) < 7.15 kg/m^2^, ALMI(BMI) < 0.483, and T-score: [(ALMI(h^2^) measured)-(2.08 + 0.183*BMI)]/0.72] − the LLTM mass prevalence was 17.37%; 8.47, 14.8 and 12.71%. respectively. This study showed that the current cut-offs for LLTM as criteria for sarcopenia diagnosis are not adapted to the obese population. Although the new “static” cut-offs appeared to be more adapted, a “dynamic” cut-off for ALMI(h^2^) that took into account the BMI and thus the obesity severity appeared even more relevant.

## Introduction

Aging is associated with a gradual change in body composition characterized by a decrease in muscle mass and a relative increase in fat mass (FM). This has mostly been documented in normal-weight individuals^1–4^. The decrease in muscle mass can lead to sarcopenia, a syndrome characterized by the progressive and generalized loss of skeletal muscle mass, with aging inducing weakness or poor physical performance^5^. Sarcopenia has serious consequences for health, such as physical disability, poor quality of life, increased risk of falls and fractures, and institutionalization, which all heighten the mortality risk^5–7^. The European Working Group on Sarcopenia in Older People (EWGSOP) recently published an updated consensus definition using a diagnostic algorithm (EWGSOP2); it is based on measurements of muscle strength by handgrip strength and physical performance by gait speed^8^. A concomitant evaluation of muscle quantity or mass is also recommended, that can be estimated by variety of techniques such as magnetic resonance imaging (MRI) and computed tomography (CT) that are considered to be the gold standards methods. However, these tools are not commonly used in primary care because of high equipment costs and dual-energy X-ray absorptiometry (DXA), or bioelectrical impedance analysis (BIA) as an alternative second choice, are recommended to assessed body composition^8,9^.

Although sarcopenia in most individuals is largely attributable to aging (primary), it may be secondary to a systemic disease, physical inactivity or the inadequate intake of energy or protein^8^, and it has been associated with obesity. Indeed, obesity, which is characterized by excessive FM as a percentage of body weight associated with a relative increase in lean tissue mass (LTM)^10^, may exacerbate sarcopenia through fat infiltration into muscle, thereby lowering physical function^11^. The combination of these two unfavorable situations has been called “sarcopenic obesity”^12^, although despite recently increased interest, the prevalence of sarcopenic obesity remains unclear. This was pointed out by Batsis et al.^13^, who reported that the sarcopenic obesity prevalence for women ranged from 3.6 to 94% when eight separate definitions of skeletal muscle mass were applied to a representative NHANES sample of non-institutionalized individuals with obesity aged 60 years or older^13^. This wide variability can be explained by several factors, notably the cut-offs to define obesity and sarcopenia, suggesting the need for consensual criteria^14^. Recently, a consensus paper on sarcopenic obesity was published, underlining the current limited knowledge and encouraging efforts to improve the methods of identifying and treating affected patients^9^ for use in routine care settings^15^.

It is likely that the cut-offs for low LTM^5,8,16^ used in the general population are not transposable to patients with obesity. Although few data are available^17^, it appears that subjects with obesity present a specific change in body composition with aging. For example, we recently reported that although women with obesity showed a localized redistribution of body FM and LTM with aging^18^, none presented sarcopenia when the cut-offs for appendicular lean mass index [appendicular lean mass/height^2^: ALMI(h^2^) < 5.5 kg/m^2^ for women] defined by the EWGSOP2 were applied^8^. Two questions arose from this observation: (i) Do patients with obesity develop sarcopenia? and (ii) Are the cut-offs currently used appropriate for this population? The reduction in the ALMI(h^2^) observed in these patients, which has been validated as a criterion for identifying the sarcopenia of aging^18^, points more to the possibility that the currently used cut-offs may be inappropriate. This also confirms the message from the EWGSOP2 and the ESPEN (European Society for Clinical Nutrition and Metabolism)/EASO (European Association for the Study of Obesity) statement, which underscores that sarcopenic obesity is a distinct condition^8^ that needs a precise definition and adapted cut-offs^9,19^.

The three aims of this study were therefore to: (i) determine the prevalence of low LTM − a criterion of sarcopenia diagnosis − in older women with obesity applying commonly used cut-offs for muscle mass, (ii) build a new algorithm redefining low LTM in obese women with a new ALMI cut-off based on data from young French women and taking into account their body mass index, and (iii) re-determine the prevalence of low LTM by applying this new cut-off in a population of obese women 60 years or older.

## Materials and methods

### Participants

Women with obesity as defined by a body mass index (BMI) ≥ 30 kg/m^2,20^ were recruited consecutively between December 2010 and September 2020 in the Nutrition Clinic of the University Hospital of Montpellier, France, where they had been referred for metabolic and physical assessment of obesity or a medical check-up before bariatric surgery. We have focused our research on female patients because they constitute the majority of patients treated in our department and none had undergone bariatric surgery. The patients were subdivided into two groups according to age: young patients from 18 to 35 years old and patients 60 years or older.

Young premenopausal women (age range 18–35 years) with normal weight constituted the young healthy control group. These participants were recruited by local advertisement or from friends of patients who had agreed to participate. Individuals in the control group reported no history of obesity (BMI from 18 to 24.9 kg/m^2^), diabetes mellitus, hypertension or dyslipidemia.

All investigations and measurements are described in detail elsewhere^18^ and were performed in fasting conditions in the morning (8:30–10 a.m.). The exclusion criteria were pregnancy, acute medical treatment, and any physical handicap (amputation, neurological lesion, orthopedic prosthesis) that might interfere with the body composition measurement. Moreover, participants with a body weight > 190 kg or height ≥ 192.5 cm were also excluded due to the limitations of the densitometry device. The medical history and menopausal status, when relevant, were obtained by questionnaire. The histories of smoking status and diabetes mellitus, as well as current medications, were also recorded.

For all participants, the standing height was measured with a stadiometer to the nearest 0.1 cm. The height and weight were measured with participants wearing light clothing and no shoes, and the BMI was calculated as the weight in kilograms divided by the square of the height in meters (kg/m^2^). The waist circumference was recorded to the nearest 0.1 cm midway between the last rib and the crest of the ileum using a non-stretch tape measure. These two measurements were performed only in the subjects with obesity.

Comorbidities were defined according to the usual definitions:

Type 2 diabetes was defined as HbA1c ≥ 6.5%, and/or fasting glycemia ≥ 7 mmol/L, and/or antidiabetic treatment^21^.

Arterial hypertension (HTA) was defined as systolic blood pressure > 140 mmHg, and/or diastolic blood pressure > 90 mmHg, and/or use of anti-hypertensive medications^22^.

All the data on demographics and clinical characteristics were collected by the endocrinologist referents for obesity care.

### Participant consent

All participants gave written informed consent. The study was performed according to the principles of the Declaration of Helsinki and was approved by the local ethics committee (CPP Sud-Méditerranée IV, Montpellier, France). The data of all participants were entered into a registry and included anthropometric, clinical and biological information and body composition determination (NDC-2009-1052). This study was performed in accordance with the ethical standards laid down in the 1964 Declaration of Helsinki and its later amendments.

### Body composition determination

The procedure is described elsewhere in detail^23^. The soft tissue [(FM, kg), (FM,%) and (LTM, kg)] were measured using DXA (Hologic QDR-4500A, Hologic, Inc., Waltham, MA). Data at each localized site (upper limbs, trunk, and lower limbs) were derived from the whole-body scan. The trunk was defined as the whole body excluding limbs and head. All scanning and analyses were performed by the same operator to ensure consistency after following standard quality control procedures. Quality control for DXA was checked daily by scanning a lumbar spine phantom consisting of calcium hydroxyapatite embedded in a cube of thermoplastic resin (DPA/QDR-1; Hologic x-caliber anthropometrical spine phantom). The coefficient of variation (CV) given by the manufacturer was < 1% for LTM and FM.

### Currently used cut-offs for the definition of low LTM

Appendicular lean mass (ALM; kg) was defined as the sum of the LTM of the arms and legs, as described by Heymfield et al.^24^. The ALM index was defined as ALM/height^2^ [ALMI(h^2^); kg/m^2^] to eliminate differences in ALM associated with greater height in younger adults^25^ or as ALMI/body mass index [ALMI(BMI)]. As ALMI thresholds for defining sarcopenia may vary between ethnic groups, like Asian and Caucasian^26,27^, we chose to use the most frequently adopted muscle mass cut-offs for the definition of sarcopenia for Caucasian women. First, EWGSOP2 defined two cut-offs: ALM < 15 kg and ALMI(h^2^) < 5.5 kg/m^2^, which together form the most widely accepted definition for sarcopenia^8^. Second, for the Foundation for the National Institutes of Health (FNIH), the cut-points for low LTM are ALM < 15.02 kg and ALMI(BMI) < 0.512^28^. These cut-offs were based on the analyses of several cohorts of community-dwelling older women^16^. The FNIH project chose a data-driven process, where the cut-point for low muscle mass was based on the risk of weakness and not relative to a healthy young reference population, as in the EWGSOP definitions. FNIH uses ALM with the recommendation to adjust for body mass index (BMI), low grip strength to define weakness, and low gait speed to define slowness. Third, the International Working Group on Sarcopenia (IWGS) defined sarcopenia as ALMI(h^2^) < 5.67 kg/m^2^^29^.

### Validation of low LTM cut-offs from young French non-obese women

As recommended in previous publications^8^, and because the French and American populations might differ in terms of BMI and body composition, we re-estimated the threshold values to define low LTM in order to adapt them to our local French population. We used the method proposed by Baumgartner et al.^25^ that consists of estimating the mean values of ALM and ALMIs in young adult women controls (CON) from 18 to 35 years old with normal weight (18 kg/m^2^ ≤ BMI ≤ 25 kg/m^2^). Cut-offs for low LTM were defined as values 2 standard deviations below the sex-specific means of the ALM, ALMI(h^2^) and ALMI(BMI) reference data for young adult women without obesity.

### Determination of new low LTM cut-offs from young French women with obesity

To determine the new low LTM cut-off values for patients with obesity, we adapted the method proposed by Baumgartner et al.^25^ and estimated the mean values of ALM and ALMIs in young adult women with obesity (from 18 to 35 years old with BMI > 30 kg/m^2^) rather than in healthy young women with normal BMI. Cut-offs for low LTM were defined as values 2 standard deviations below the sex-specific means of the ALM, ALMI(h^2^) and ALMI(BMI) reference data for young adult women with obesity.

Last, because it seemed likely that ALM and ALMI(h^2^) [and even ALMI(BMI)] would be dependent on BMI, we sought to define a dynamic threshold for low LTM adapted to the BMI of obese women. We therefore used a linear regression model to estimate an equation for this population of young women with obesity to determine the mean value (= expected value) of ALMI(h^2^) depending on BMI. The dynamic thresholds for low LTM were set at -2 standard deviations of the residuals of the linear model from the expected value of ALMI(h^2^) for the calculated BMI.

### Statistical analysis

Patient characteristics are described as proportions for categorical variables and as means ± standard deviations (SD) for quantitative variables. Comparisons between groups were made using ANOVA or Student’s t-test for quantitative variables or the Kruskal–Wallis or Mann–Whitney test when the distribution of variables was identified as non-Gaussian. We also compared the groups two by two using Student’s t-test or the Mann–Whitney U-test. Qualitative variables were analyzed using the Chi2 test. The Tukey–Kramer procedure accounted for the inflation of alpha risk associated with multiple comparisons. Given that any difference in body composition between the two obesity groups might be due to differences in weight and height, we used a multivariate linear regression model to calculate the adjusted means for these two covariates.

We then applied the definitions and cut-offs of low LTM for sarcopenia diagnosis to our population of older adults with obesity in order to observe the differences in estimates of low LTM prevalence obtained with these indicators. Finally, to calculate our dynamic threshold, we used a linear regression model to estimate the relationship between ALMI(h^2^) and BMI. The results of the equation allowed us to define a T-score based on the difference between the observed and expected (model-predicted) values of ALMI(h^2^), divided by the standard deviation of the residuals of the linear model.

$${\text{T}} - {\text{score }} = \, \left[ {\left( {{\text{ALMI}}\left( {{\text{h}}^{2} } \right)\;{\text{ measured}}} \right) \, - \, \left( {{\text{ALMI}}\left( {{\text{h}}^{2} } \right) \, \;{\text{predict}}\;{\text{ by}}\;{\text{ the }}\;{\text{model}}} \right)} \right] \, / \, \left( {{\text{standard }}\;{\text{deviation }}\;{\text{of}}\;{\text{ the}}\;{\text{ residuals}}\;{\text{ of}}\;{\text{ the}}\;{\text{ model}}} \right)$$

This T-score test was used to quantify the severity of sarcopenia. Patient were considered to have an abnormal value if their measure was greater or lower than the prediction ± 2 Standard deviation (SD).

Statistical analyses were performed at the conventional two-tailed α level of 0.05 using SAS version 9.4 (SAS Institute, Cary, NC).

## Results

### Participant characteristics

**Figure 1:**
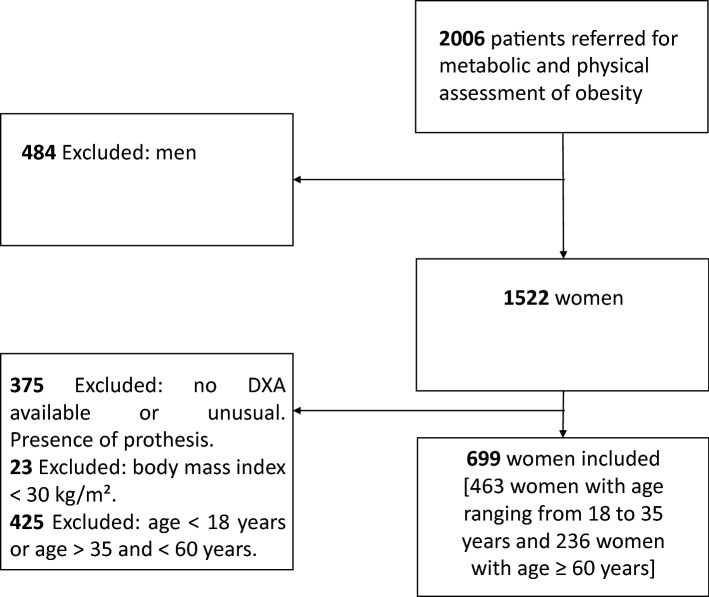
Study population with obesity.

The sample included 791 Caucasian women with an age distribution from 18 to 81.9 years. Women with obesity (n = 699) were subdivided in two subgroups according to age. Women from 18 to 35 years old constituted the “young adults with obesity group” (n = 463; mean age 26.6 ± 4.8 years) while women from 60 to 81.9 years old (n = 236; mean age 66.8 ± 4.6 years) constituted the “older adults with obesity group” (Fig. 1). Most of the participants had a long-standing history of obesity (> 5 years). Ninety-two young women with normal weight and from 18 to 35 years old constituted the control group (mean age 23.2 ± 3.2 years).

The baseline anthropometric characteristics and the comparisons between groups according to obesity status and age are summarized in Table 1. Physical activity levels were not specifically determined. Nevertheless, the control and patient groups consisted of individuals who performed only leisure physical activities for less than one hour per week. Moreover, none of the patients was enrolled in a training program on the day of inclusion.

**Table 1:** Demographic and health characteristics regarding age group and obesity status.

	Young healthy subjects	Young patients with obesity	Older patients with obesity
Number of participants (n)	92	463	236
Age (years)	23.2 ± 3.2	26.6 ± 4.8	66.8 ± 4.6
**Clinical characteristics**			
Weight (kg)	60.6 ± 7.3	109.4 ± 14.6*	100 ± 15.8*^§^
Height (m)	1.66 ± 0.06	1.64 ± 0.06	1.59 ± 0.06*^§^
Body mass index (kg/m^2^)	22 ± 2.3	40.6 ± 5.1*	39.7 ± 5.6*
Waist circumference (cm)	–	111.5 ± 14.4	114.3 ± 11.9^§^
Hip circumference (cm)	–	128.4 ± 11.4	125.1 ± 13^§^
**Comorbidities**			
HTA (number; %)	–	13 (2.8%)	166 (70.3%)^§^
Diabetes (number; %)	–	25 (5.4%)	115 (48.7%)^§^

Data are presented as the mean ± standard deviation. *HTA* arterial hypertension. Body mass index for young healthy controls ranged from 18 kg/m^2^ ≤ BMI ≤ 25 kg/m^2^ and was ≥ 30 kg/m^2^ in patients with obesity. We used the Tukey–Kramer procedure to control for the multiple comparison problem. * denotes a significant difference compared to the 18–35 normal-weight women group. ^§^ denotes a significant difference compared to 18–35 young women with obesity group.

**Figure 2:**
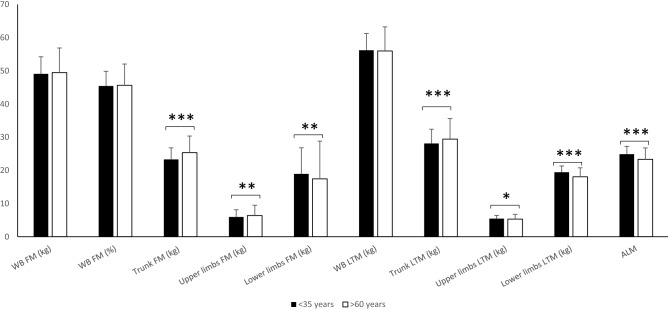
Comparision of fat mass and lean tissue mass adjusted on weight and height between young and older women with obesity. Data are presented as mean ± SD. WB: whole body; FM: fat mass; LTM: lean tissue mass; ALM: appendicular lean mass * indicates a significant difference between the two groups for *p* < 0.05, ** for *p* < 0.01 and *** for *p* < 0.001 .

The whole-body and localized LTM and FM are presented in Table 2. For all body composition parameters and low LTM indices [ALM and ALMI(h^2^)], the control group presented systematically lower [ALMI(BMI) excepted] values than the women with obesity, regardless of age. Young women with obesity presented higher whole-body and lower limb FM, and higher whole-body, trunk, upper limb and lower limb LTM. Further, low LTM indices [ALMI(BMI) excepted] were higher than those of the older women with obesity. As the difference in LTM and FM between the two obesity subgroups could have been due to weight and height differences, adjustment on these two covariables was performed (Fig. 2). Although whole-body FM and whole-body LTM were comparable between the two groups, trunk FM, upper limb FM, and trunk LTM remained lower in the young women with obesity compared with the older women. Conversely, upper limb LTM, lower limb LTM and ALM were higher.

**Table 2:** Whole body, truncal and appendicular fat mass and lean tissue mass in women regarding age group and obesity status.

	Young healthy individuals	Young patients with obesity	Older patients with obesity
Number of participants (n)	92	463	236
**Fat mass**			
Whole body (kg)	17.6 ± 4.2^§^	50.6 ± 9.3*	46.4 ± 10.7*^§^
Whole body (%)	28.9 ± 4.7^§^	45.5 ± 4.0*	45.4 ± 4.4*
Trunk (kg)	6.6 ± 1.9	24.1 ± 5.2*	23.8 ± 5.6*
Upper limbs (kg)	2.1 ± 0.6	6.1 ± 1.4*	6.1 ± 2.8*
Lower limbs (kg)	8.1 ± 2.0	19.6 ± 6.2*	16.1 ± 9.1*^§^
**Lean tissue mass**			
Whole body (kg)	40.8 ± 4.6	57.8 ± 7.4*	52.9 ± 6.8*^§^
Trunk (kg)	20.2 ± 2.4	28.9 ± 4.4*	27.8 ± 4.7*^§^
Upper limbs (kg)	4.0 ± 0.6	5.6 ± 1.0*	5.0 ± 0.9*^§^
Lower limbs (kg)	13.6 ± 1.9	20.1 ± 2.9*	16.8 ± 3.0*^§^
**Sarcopenic index**			
ALM (kg)	17.6 ± 2.3	25.6 ± 3.6*	21.8 ± 3.5*^§^
ALMI(h^2^) (kg/m^2^)	6.4 ± 0.7	9.5 ± 1.2*	8.7 ± 1.2*^§^
ALMI(BMI)	0.80 ± 0.1	0.64 ± 0.08*	0.55 ± 0.07*^§^

Data are presented as the mean ± SD. Body mass index for young healthy controls ranged from 18 kg/m^2^ ≤ BMI ≤ 25 kg/m^2^ and was > 30 kg/m^2^ in patients with obesity. *ALM* appendicular lean mass; *ALMI* appendicular lean mass index [ALMI(h^2^): ALM/height^2^ and ALMI(BMI): ALM/BMI)]; *BMI* body mass index (weight/height^2^). We used the Tukey–Kramer procedure to control for the multiple comparison problem. *denotes a significant difference compared to the 18–35 normal-weight women group. ^§^ denotes a significant difference compared to 18–35 women with obesity group.

### Prevalence of low LTM in older women with obesity with the currently used cut-offs

The prevalence of low LTM in the older women with obesity was estimated according to the different definitions retained for Caucasian women^8,28,29^ and is presented in Table 3. Our results indicated a wide distribution of low LTM prevalence in these older women, ranging from 0% according to EWGSOP2 and IWGS to 29.2% according to FNIH.

**Table 3:** Prevalence of low ALM-ALMI(h^2^)-ALM/BMI in older patients with obesity using different currently used and newly determined cut-offs.

Reference used	Diagnostic criteria	Cut-offs points	Prevalence of low ALM-ALMI in older obese patients, n (%)
**Cut-offs from previous studies**			
EWGSOP2	ALM	< 15 kg	3 (1.27%)
EWGSOP2	ALMI(h^2^)	< 5.5 kg/m^2^	0 (0%)
IWGS	ALMI(h^2^)	< 5.67 kg/m^2^	0 (0%)
FNIH	ALMI(BMI)	< 0.512	69 (29.2%)
**Cut-offs calculated from our population (18–35 years)**			
**Validation of thresholds currently used from young non-obese women data**			
	ALM (-2 SD)	12.88 kg	0 (0%)
	ALMI(h^2^) (-2 SD)	< 5.0 kg/m^2^	0 (0%)
	ALMI(BMI) (-2 SD)	< 0.614	196 (83.1%)
**Determination of new thresholds from young obese women data**			
	ALM (-2 SD)	< 18.51 kg	41 (17.37%)
	ALMI(h^2^) (-2 SD)	< 7.15 kg/m^2^	20 (8.47%)
	ALMI(BMI) (-2 SD)	< 0.483	35 (14.8%)
**Dynamic thresholds based on our new T-score**			
	T-score = [ALMI(h^2^)—(2.08 + 0.183*BMI)] / 0.72	< 2.0	30 (12.71%)

Data are presented as number (percentage; %) of older patients with obesity. *EWGSOP2* European Working Group on Sarcopenia in Older People; *IWGS* the International Working Group on Sarcopenia; *FNIH* The Foundation for the National Institutes of Health; *ALM* appendicular lean mass; *ALMI* appendicular lean mass index [ALMI(h^2^): ALM/height^2^ and ALMI(BMI): ALM/BMI)]; *BMI* body mass index (weight/height^2^). *SD* standard deviation.

### Prevalence of low LTM in older women with obesity using cut-offs calculated from young French women with normal body weight

We next used the data from the young normal-weight women to validate the current cut-offs for ALM, ALMI(h^2^) and ALMI(BMI) (Table 3). No older patients with obesity presented low LTM when the cut-offs for ALM (12.88 kg) and ALMI(h^2^) (5.0 kg/m^2^) were calculated from normal-weight young women, while the prevalence was 83.1% when ALMI(BMI) (< 0.614) was used.

### Prevalence of low LTM in older women with obesity using cut-offs calculated from young French women with obesity

In these same older women with obesity, the low LTM prevalence was, respectively, 17.37 and 8.47% when the ALM (18.51 kg) and ALMI(h^2^) (< 7.15 kg/m^2^) cut-offs were calculated from young women with obesity, whereas it was 14.8% when ALMI(BMI) (< 0.483) was used.

**Figure 3:**
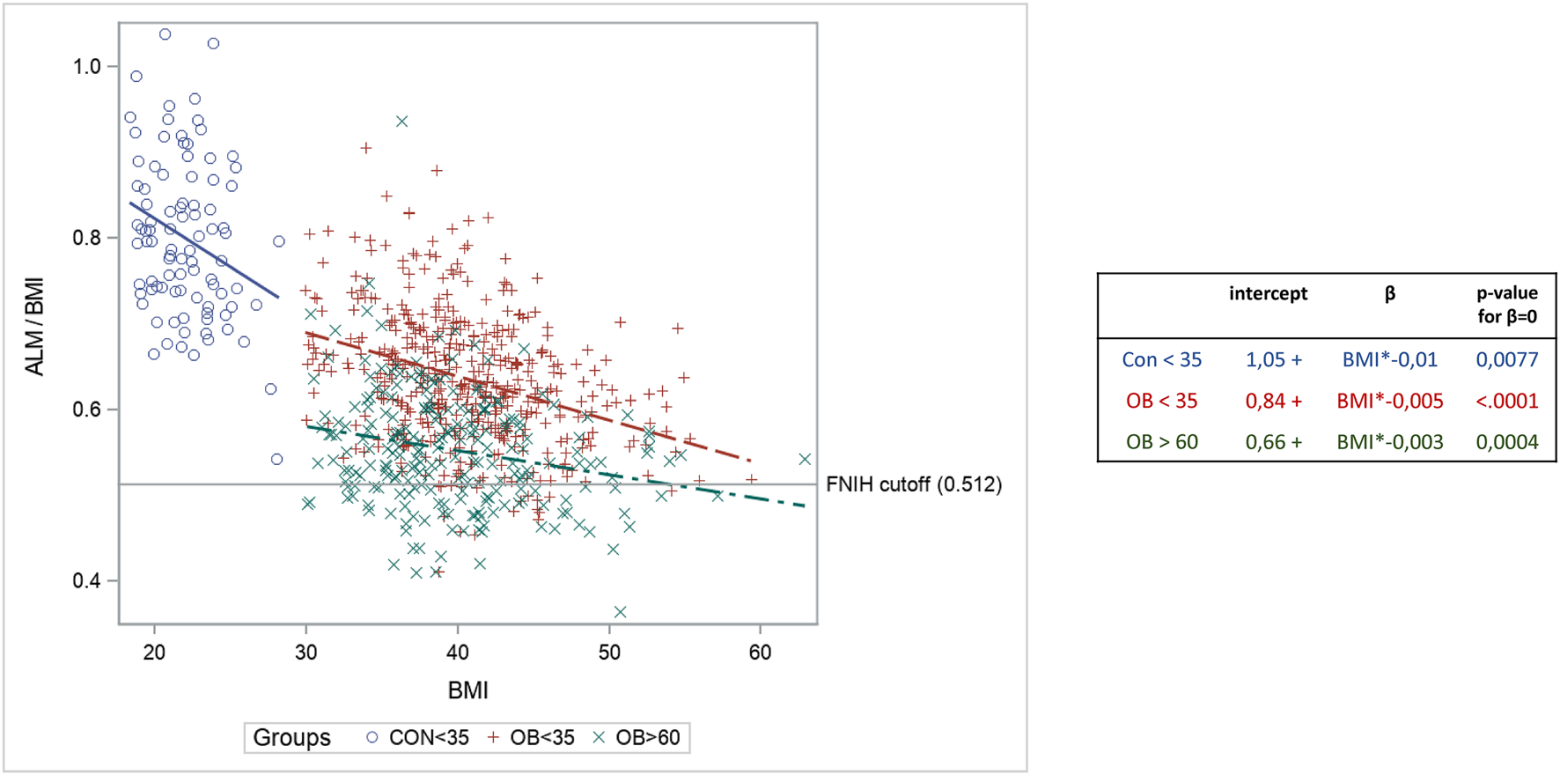
Regression curve of ALB/BMI accordibg to BMI values and obesity status. ALM: appendicular lean mass (sum of the lean soft tissue mass for the arms and legs), BMI: body mass index; FNIH: foundation for the national institutes of health. CON: young women with normal weight; OB ≤ 35: young women (18–35 years) with obesity; OB > 60: older women (> 60 years) with obesity.

To understand this wide range of low LTM prevalence, we studied the distribution of ALMI(BMI) according to BMI in each group (Fig. 3). These data confirmed the reduction in ALM with age in the patients with obesity, because whatever the BMI, the older women presented lower ALMI(BMI) values than the young women. Nevertheless, the regression curve also showed that ALMI(BMI) was greatly influenced by BMI. Further, the FNIH cut-off used for ALMI(BMI), which is a fixed value, tended to over-detect low LTM in patients with severe obesity (regression slope negative and significantly different from 0 in all three groups).

**Figure 4:**
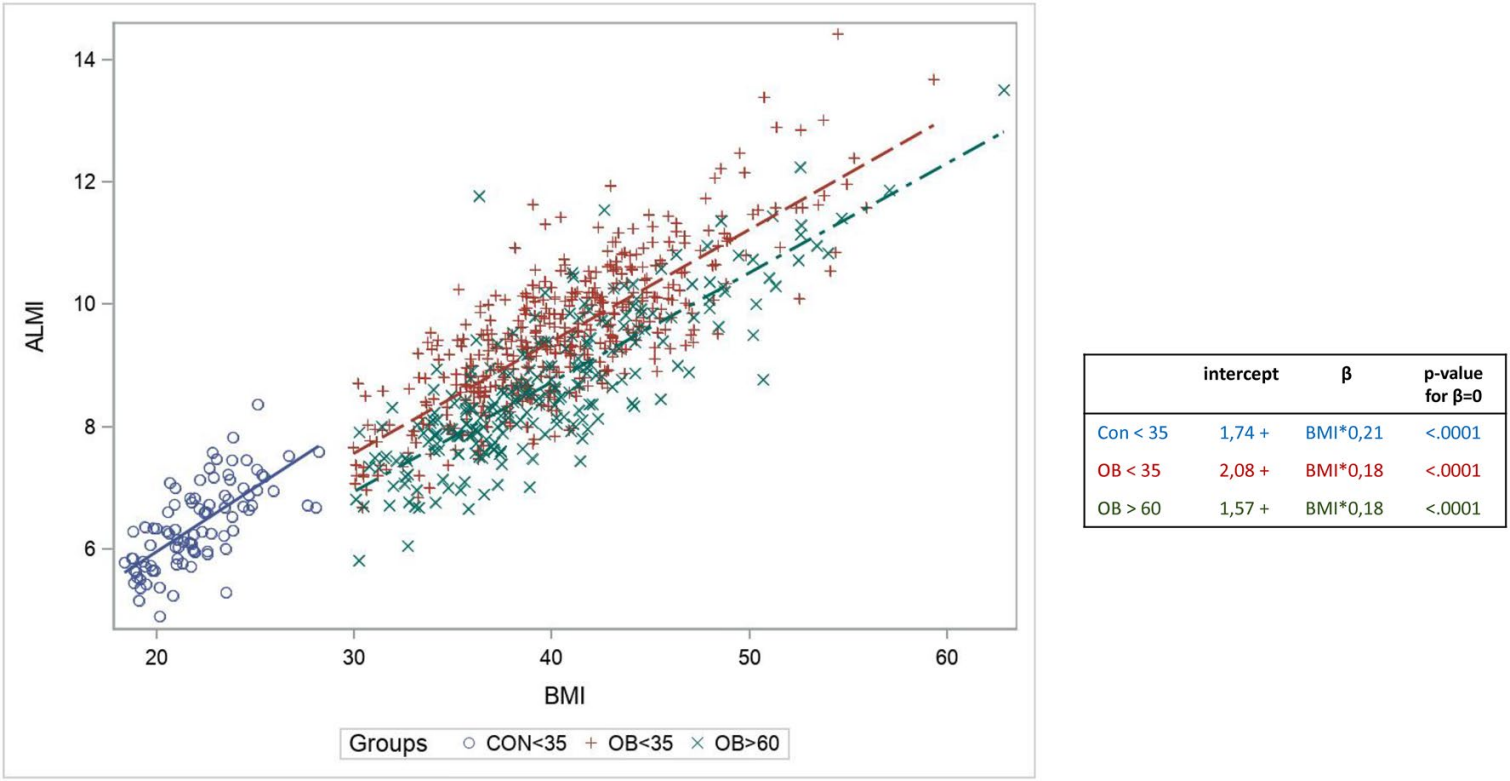
Linear regression explaining the ALMI of young obsese patients according to the BMI. ALMi: appendicular lean mass (sum if the lean soft tissue mass for the arms and legs)/height^2^, BMI: body mass index. CON: young women with normal weight; OB ≤ 35: young women (18–35 years) with obesity; OB > 60: older women (> 60 years) with obesity. Patients with ALMI < 0.6428224 + 0.18281*patient’s BMI can therefore be considered to be sarcopenic.

The relationship between ALMI(h^2^) and BMI in the three groups is illustrated in Fig. 4. A linear relationship between ALMI(h^2^) and BMI can be observed in each of the three groups, with the apparent continuity of the regression line for “non-obese youth” and “obese youth.” The line for the “older adults with obesity” group was almost parallel to that of the “young adults with obesity” but with a lower expected value of ALMI(h^2^) for a given BMI. The modeled ALMI(h^2^) values according to BMI in the young obese women resulted in the determination of the following equation for the expected normal value of ALMI(h^2^): *ALMI(h*^*2*^*) predict* = *2.08* + 0.183 * BMI, with the normal values of ALMI(h^2^) being included in the interval “ALMI(h^2^) predict  ± 1.44” (± 2 SD of the residual of the model). Consequently, older patients with obesity with ALMI(h^2^) < 0.64 + 0.183* *patient’s BMI* were defined as low LTM.

For more complete information about the severity of low LTM, we used this equation to calculate a “T-score” for each patient, which directly represented the deviation from the reference values:

$${\text{T - score }} = \, [({\text{ALMI}}\left( {{\text{h}}^{2} } \right){\text{measured}}) \, - \, \left( {2.08 \, + \, 0.183 \, *{\text{ BMI}}} \right)] \, / \, 0.72$$

**Figure 5:**
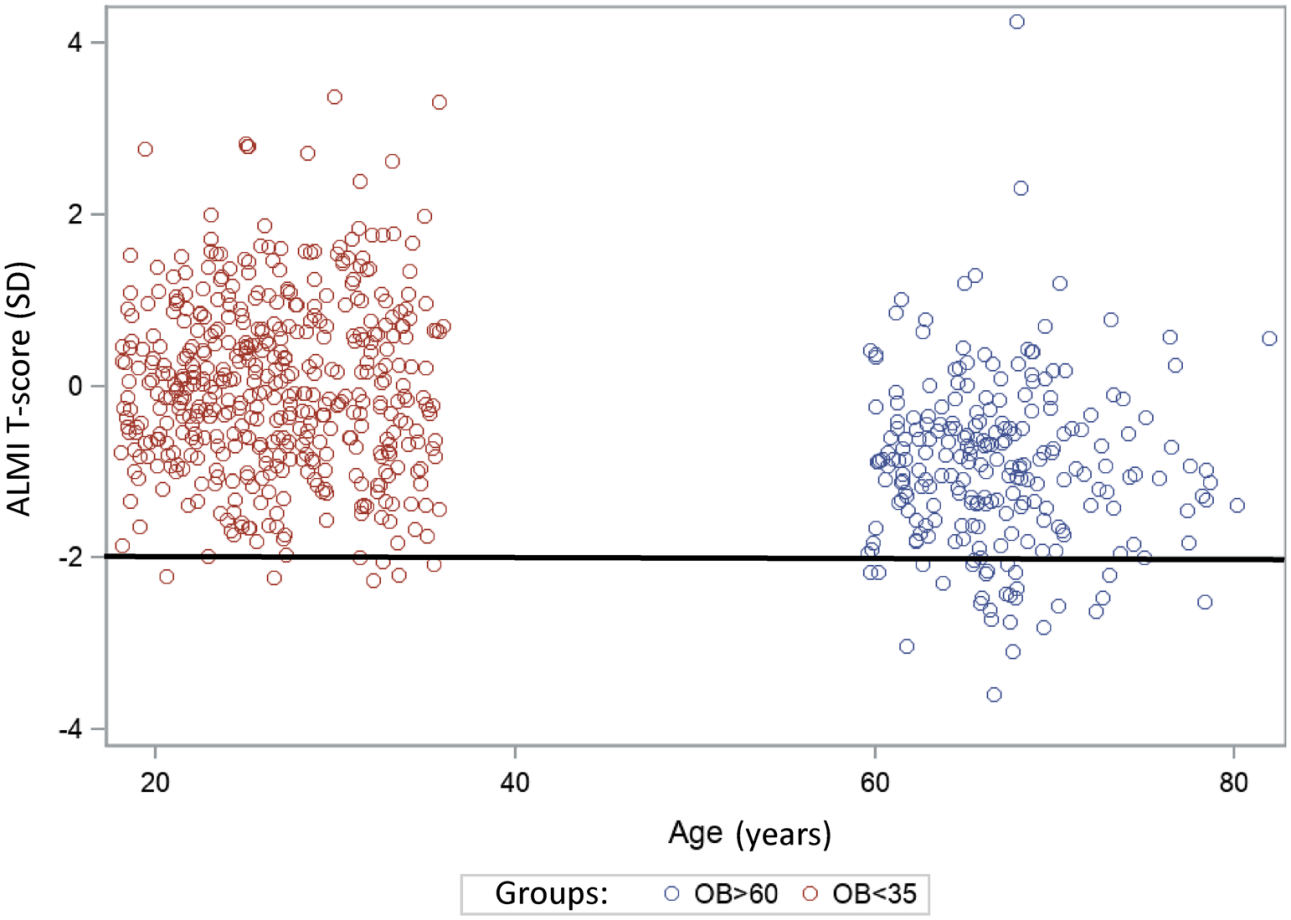
ALMI T-score determined in young and older women with obesity. ALMI: appendicular lean mass index (sum of the lean soft tissue mass for the arms and legs)/height^2^; SD: standard deviation. T-score was calculated as follows: [(ALMi measured) − (ALMI predict)]/(standard deviation) with ALMI predict = 2.07996 + 0.1828*BMI and SD = 0.7180668. Patients were considered sarcopenic when ALMI T-score <  − 2DS.

Thus, patients with a T-score lower than -2 SD were considered to have low LTM. When this new cut-off value was applied to our population, the low LTM prevalence in the older patients with obesity was 12.71% (n = 30). The T-scores for the young and older adults with obesity are shown in Fig. 5.

## Discussion

This study described the wide variation in low LTM values among older women with obesity, depending on the cut-offs used. However, although ALMI(h^2^) and ALMI(BMI) were lower in the older than in the younger obese women, the older women with obesity presented no or few cases of lower LTM − a parameter included in the definition of sarcopenia − for most of these current cut-offs. These findings confirmed that the current cut-offs used to diagnose low LTM in the general older population are not adapted to French older women with obesity. The development of new cut-offs, calculated from young obese women with the same disease, may be better adapted.

The body composition changes with aging have been well-described in the normal-weight population and are primarily characterized by a decrease in muscle mass and an increase in FM^1,3,4^. However, the model of change in subjects with obesity remains insufficiently known. To our knowledge, only one study using NHANES data tried to model the age-related changes in segmental body composition (SBC) according to BMI (from normal weight to obese). These authors assumed that there is a constant BMI-related difference in SBC in all age classes^17^ and thus that all the BMI categories share the same trends for aging. On the basis of this study and other recently published data^18^, our group found that although whole-body LTM and FM evaluated by DXA were relatively constant with aging, individuals with obesity presented a localized redistribution of these two components. More specifically, the older obese group presented lower LTM and FM at appendicular sites, particularly in the lower limbs, and higher LTM and FM at the central body compared to the younger obese group^18^.

Part of this body composition variation with age could be attributed to the change in the anthropometric parameters, as the weight and height were lower in the older adults with obesity. This was due not only to the age-related decline in stature, but also to a generational gap (a trend for a steady increase in the stature of European women in the last 80 years has been reported^30,31^). However, the adjustment on these two covariables did not modify our results, suggesting a reduction in LTM with age in the obese population. Despite our finding that LTM was lower in the older obese women compared to the younger obese group, none of the patients presented with low skeletal muscle mass when the various currently used cut-off definitions were applied, including those from EWGSOP2 [ALMI(h^2^) < 5.5 kg/m^2^]^8^ and IWGS [ALMI(h^2^) < 5.67 kg/m^2^]^29^. We assumed that the cut-offs used in our study would be fully applicable to Caucasian women and therefore that their use would not constitute a methodological bias. Further, ethnicity has been acknowledged to be an important explanation for the big gaps in cut-off points among different study populations^14,32,33^ . To confirm our assumption, we recalculated the cut-offs using the method of Baumgartner et al.^25^, with -2 SD of the reference values from young French normal-weight women of a similar ethnic group. The cut-off for ALMI(h^2^) was 5.0 kg/m^2^, which was roughly comparable to those reported by EWGSOP2 and IWGS^8,29^, the Rosetta study (< 5.5 kg/m^2^)^25^, the Health ABC study (5.67 kg/m^2^)^34^, and in Italy (4.82 kg/m^2^)^30^. Our results confirmed that the currently used cut-offs in this study are applicable to our regional population. However, none of our older subjects apparently suffered from low LTM, which should prompt us to question the reality or myth of the development of this disease in this specific population. Only our results concerning ALMI(BMI) (< 0.614) seemed to overestimate the prevalence of low LTM compared to the cut-off provided by FNIH (< 0.512), and this may be attributed to the different BMI of the reference population.

As mentioned above, the reduction in LTM, ALM and ALMI(h^2^) points more to an inability of the commonly used cut-offs to identify subjects with low LTM. The higher LTM and ALMI(h^2^) values in the older patients compared to those of the normal-weight controls reinforced the crucial need for the definition of new thresholds for sarcopenic obesity adapted to this population by taking into account the specificity of the anthropometric characteristics of patients with obesity^18^.

In this context, Batsis et al.^14^ recommended the development of obese population-specific thresholds to identify older adults who have sarcopenia in terms of muscle mass. In this study, we calculated new cut-offs for the first time from a large group of representative young French women with obesity. As these young patients presented higher total and appendicular LTM compared with healthy controls, a higher ALMI(h^2^) cut-off was obtained (i.e., 7.51 kg/m^2^), necessarily leading to an increase of the prevalence of low LTM to 8.47%. The question that remains is the following: Is this new prevalence of the same magnitude as that found in older normal-weight subjects? It is difficult to precisely answer this as many factors influence low LTM onset, progression and diagnosis^35^. Moreover, it was demonstrated that the prevalence varies with age ^12^, methods of evaluation (DXA or bioelectrical impedance)^36^, and the cut-offs used^14^. A meta-analysis of 19 studies demonstrated an overall low LTM prevalence of 17% of older Brazilians aged ≥ 60 years^37^. In a multicenter study of healthy older women (mean age 74.4 years) in five European countries (including France), the prevalence of low muscle mass was varied between 0.3 and 14.9% according to the cut-off used for ALMI/height^2^ (11.4% for ALMI(h^2^) < 5.45 kg/m^2^)^38^. Using the same diagnostic criteria, this prevalence increase in geriatric outpatients (mean age 81.1 years) from 2.4 to 22.8% (22% for ALMI/(h^2^) < 5.45 kg/m^2^)^38^. Park et al.^39^ reported that the prevalence of low LTM using different cut-offs for ALMI(h^2^) ranged from 18.5 to 33.3% in postmenopausal women with ages ranging from 60 to 70 years. Finally, Baumgartner et al.^25^ reported that the low LTM prevalence defined as ALMI < 5.45 kg/m^2^ was 23.1% in people under 70 years, but it increased with age to reach 30% to 50% in women older than 80 years ^25,40,41^. When older subjects with obesity were specifically studied, the prevalence of low LTM using the current cut-offs was systematically lower than in the general (non-obese) population. In a study using the EWGSOP2 criteria and an obesity definition from the fat percentile method, Bahat et al.^42^ recently reported a prevalence of approximately 4% of sarcopenia obesity in obese older subjects (60–99 years) when skeletal muscle mass (SMM) evaluated by BIA was adjusted by BMI and 0.2% when SMM was adjusted by height^2^. Moreover, these authors underlined that among sarcopenic patients, obesity may have a protective effect against the limitations of some functional measures, indicating a probable protective effect of obesity in sarcopenic individuals^42^. Using cut-offs comparable to those used in our study, Zoico et al.^43^ observed than 12% of these participants, all older Italian women, presented low LTM and obesity concomitantly. Finally, in a recent meta-analysis that included 40 studies, Gao et al.^44^ found that the global prevalence of sarcopenic obesity was 15% (14% for women and 10% for men), when muscle mass alone was used for the definition. Interestingly there were no significant differences in the prevalence of sarcopenic obesity among studies using different criteria for obesity definition^44^.

Although our results do not seem to be complete outliers, establishing new cut-offs for low LTM diagnosis in patients with obesity is not easy, and the choice of ALMI (h^2^ or BMI) -2 SD from young patients merits discussion. There is an ongoing debate about the preferred adjustment [ALMI(h^2^), ALM/weight or ALMI(BMI)] and whether the same method can be used for all populations^45^. Also, it is unknown whether patients with obesity have the same time course for muscle loss as normal-weight subjects. We demonstrated that the bone loss with aging is reduced in women with obesity compared with normal-weight patients^46^. Also, as muscle mass was found to be independently associated with bone mass^46^ and its loss often accompanies bone loss^47^, it is probable that the loss of muscle mass was also reduced in these patients. Conversely to our hypothesis, a very recent study reported that the difference in ALMI(h^2^) between premenopausal (6.6 kg/m^2^) and late postmenopausal (6.1 kg/m^2^) non-obese women was also around 8%^39^. In a non-obese population, Kyle et al.^3^ reported a lower reduction of 3.4% for ALMI(h^2^) in women 60 to 74 years old compared to women 18 to 34 years old. Nevertheless, their strict selection of participants without mobility problems and a relatively high practice of regular physical exercise may explain the limited loss of LTM. In our study, it is probable that the higher body weight of the older women with obesity partially masked the reduction in LTM. Only a prospective study concomitantly comparing pre- and postmenopausal women with and without obesity would allow us to draw conclusions, but to our knowledge this type of study has not been done.

As previously reported^14,38^, our study highlighted that the prevalence of low LTM in patients with obesity in terms of muscle mass is highly dependent on the set of diagnostic criteria that is applied, with values ranging from 0 to 84.7%. We must keep in mind that, like the currently used cut-offs, the “static” cut-offs calculated from the young obese population in our study may have similar limitations and simply shift the problem between obesity levels. This was suggested by the observation that the highest prevalence was observed when the criteria were related to BMI [i.e., ALMI(BMI)]. It is thus probable that these new cut-offs do not identify the low LTM prevalence in the same fashion according to obesity severity. The prevalence of low LTM in patients with obesity is then gradually underestimated by ALM and ALMI based on BMI, while it is overestimated by ALM/BMI. These biases were also observed in the control group (see Fig. 2). To limit the likely effect of BMI in the obese population, we calculated a dynamic threshold for low LTM adapted to BMI. This new cut-off, calculated using the absolute ALMI(h^2^) values or measured directly by the T-score − which can easily be used by the medical community − identified low LTM in 12.71% (n = 30) of the older women with obesity. Last, the low LTM prevalence obtained with this new cut-off was situated between the prevalence obtained from ALMI(h^2^), 8.47%, and ALM(BMI), 14.0%, calculated from the reference values of the young women with obesity. It is clear that this dynamic cut-off adapted to BMI presents a clear advantage compared to the static values because it is adapted to all types of obesity severity. Moreover, this dynamic threshold for ALMI(h^2^) may also be used in non-obese populations to avoid a potential underdiagnosis in the highest “normal” BMI or for overweight subjects. Nevertheless, to definitively conclude that this dynamic cut-off is superior to static cut-offs, a stronger association with adverse outcomes such as decreased functionality or physical performance will need to be demonstrated.

### Study limitations

In interpreting the study findings, some limitations should be considered. The main limitation is the cross-sectional design that may have introduced generational bias, such as weight and height variations. Nevertheless, adjustment on these two covariables did not deeply influence the results. In addition, although it was demonstrated that the parameters reflecting lean body mass changed at a faster rate after 60 years^3^, the relatively limited number of 75- to 80-year-old patients in our study, who generally have the highest prevalence of sarcopenia in terms of muscle mass ^25,40,41^, might limit the scope of our study to the investigated age group (i.e., 60–75 years). In the current study, it should also be noted that only muscle mass was evaluated, which is less associated with functional decline and other adverse outcomes than muscle strength decrease^48^. In addition, conversely to computed tomography, DXA, which is the gold standard for analyzing LTM, is unable to directly measure muscle mass and muscle fat infiltration, which reflects muscle quality^49^. Although a new ALMI (h^2^) cut-off was defined in this study, it should be used with caution. As Delmonico et al.^50^ demonstrated, this index has limited applicability for subjects with obesity because it underestimates sarcopenia. The appendicular LTM normalized for body weight or BMI may be more adapted in the obese population^8,9,42^. Finally, we chose the methodology most often used by the WHO^20^, which defines obesity as BMI ≥ 30 kg/m^2^, as recommended by the recently published consensus statement for criteria for sarcopenic obesity^9^. Nevertheless, this consensus underlines “that future research should aim at defining the best cut points to be considered in research and clinical practice concerning sarcopenic obesity.” Threshold methodologies of body fat percentage (> 41% in females)^41^ or fat percentile above the 60th percentile^43^ seem to be of interest in this context for several authors^42,43,51^. It will be interesting to evaluate whether the prevalence of low LTM varies according to the BMI or fat percentage definition for obesity.

### Study strengths

To our knowledge, this is the first study that has sought to generate new adapted cut-offs of low LTM in older obese patients to help diagnose sarcopenic obesity in terms of muscle mass. These new cut-offs may be considered as robust because they were calculated from a large group of young obese women (n = 463) from the same country with the same disease. Moreover, another strength of our cut-offs is their potential utility in clinical practice, as they are simple and easily applicable. In addition, the development of a T-score (comparison with a young reference population) fully meets the expectations of the ESPEN/EASO consensus statement^9^.

## Conclusion

This study clearly showed that the current cut-offs used for low LTM diagnosis in the general population are not adapted to older women with obesity. Although ALMI(h^2^) was lower than in the younger obese population, no older women with obesity were diagnosed as low LTM. The development of new “static” cut-offs, such as ALMI(h^2^) calculated from young obese women with the same disease, may be better adapted. Moreover, the definition of a “dynamic” cut-off of ALMI(h^2^) that takes into account the severity of obesity and is adapted to the patient’s BMI may be even more relevant. However, before this new criterion is implemented in clinical routine, it will be necessary to determine its clinical interest and, in particular, whether it is correlated with muscle strength and physical disabilities.

## Data Availability

The datasets generated and/or analyzed during the current study are available from the corresponding author on reasonable request.
